# Characterizing the pH-Dependent Release Kinetics of Food-Grade Spray Drying Encapsulated Iron Microcapsules for Food Fortification

**DOI:** 10.1007/s11947-017-2022-0

**Published:** 2017-11-15

**Authors:** Anubhav Pratap Singh, Juveria Siddiqui, Levente L. Diosady

**Affiliations:** 1 Food Nutrition and Health Program, Faculty of Land and Food Systems, University of British Columbia, 213-2205 East Mall, Vancouver, BC V6T 1Z4 Canada; 2 Department of Chemical Engineering & Applied Chemistry, University of Toronto, 200 College Street, Toronto, ON M5T 3A1 Canada

**Keywords:** Iron microcapsules, Spray drying, Microencapsulation, Release kinetics, Food fortification

## Abstract

Iron deficiency is the primary cause of many widespread nutritional diseases including anemia, pregnancy complications, and infant mortality. Release kinetics of iron premixes to be mixed with food items like salt, rice, and tea is a key research objective of many globally active iron fortification efforts. Iron release kinetics of microcapsules of two reverse-enteric coating materials (chitosan and Eudragit EPO) encapsulating various amounts of ferrous sulfate (10–40% of total other solids) were done at three pH values (1, 4, 7) for 2 hours. Chitosan and Eudragit microcapsules contained 2.8–5.3% (*w*/*w*) and 1.7–9.6% (*w*/*w*) iron, respectively, depicting higher iron loading capacity of Eudragit microcapsules. More than 90% iron was released from most samples within 30 min under stomach conditions (pH 1) and less than 15% iron was released in 2 h under ambient conditions (pH 7), showing suitability of both chitosan and Eudragit EPO as reverse-enteric coatings for iron encapsulation. In terms of reverse-enteric behavior (RE), Eudragit EPO (RE = 2.4) was found to be slightly better than chitosan, suggesting the use of fillers in future research. Higuchi model and Hixson-Crowell model were found to best fit the data, suggesting a transport phenomenon governed by both (a) the diffusion process through the coating material and (b) the dissolution phenomenon resulting in decrease in size of the capsules. Results from this study shall provide guidance for technology development aspects of various food fortification initiatives and an understanding of the iron release from these fortificants during the food preparation and digestion stages.

## Introduction

Iron deficiency (one of the most common and most widespread nutritional disorders) affects more than two billion people worldwide, with South East Asian countries, including India and Pakistan, being worst affected. In these countries,  women and children from even affluent families and upbringings are often anemic (UNICEF 2008), suggesting a problem with dietary patterns. Cultural considerations involving vegetarianism and frequent fasting, exacerbated by insufficient intake of iron-rich animal food and consumption of diets deficient in protein and ascorbic acid results in low non-heme iron absorption. A diet rich in iron can cure can cure more than 50% of anemia cases. Furthermore, iron deficiency leads to a decreased absorption of iodine and vitamin A which causes major additional nutritional disorders (Diosady et al. 2002).

Iron deficiency is readily preventable and is not a common problem in most of the developed world due to access to multi-vitamin pills (WHO 2007). Unfortunately, this solution does not work in developing countries due to poor medical infrastructure and widespread poverty. Thus, policy intervention by the government is often desirable to implement various fortification strategies. Three main approaches available to target anemia are fortification, supplementation, and dietary interventions. Amongst them, food fortification is an inexpensive and an effective method to increase the intake of iron in the diet without compromising dietary customs. Several food products, including salt, rice, sugar, tea, wheat, cereals, and milk, have been fortified with iron and successful reduction of mortality has been observed in the test populations (Hurrel 2002). Direct mixing of iron into food materials is generally not feasible due to various reactions (redox reaction, iron-polyphenol complex, etc.) that can happen with food, and change in organoleptic properties rendering the food unacceptable. As a physical barrier between the food particles and the iron source could prevent unwanted interactions, its development is the subject of intensive research. Such physical barrier has been produced by our group through extrusion-based agglomeration, color masking, coating, spray drying, etc. for producing particles that mimic the appearance and size of the food particles (Diosady et al. 2002; Li et al. 2010). Microencapsulation of iron through spray drying has recently been attempted, and it has been found that the iron microparticles not only retain bioavailability but also prevent unpleasant taste, color, and odor in the food matrix (Dueik and Diosady 2016).

Spray drying is one of the mechanisms used for microencapsulating water-soluble iron salts with desired coating materials. Spray drying has reliably produced capsules less than 20 μm in size for the encapsulation of volatile flavors (Madene et al. 2006), oils (Jafari et al. 2008), microorganisms (Lian et al. 2003) vascular drugs (Vehring 2008), nasal drugs (Sun et al. 2009), and sparingly soluble inorganic nutrients (Oneda and Re 2003), using generally recognized as safe (GRAS) materials. Spray drying was found suitable for developing a single-step encapsulating process, simultaneously entrapping pigments, excipients, and the iron source without the complications of a multi-stepped approach (Romita et al. 2011).

Selection of appropriate coating materials depends greatly on the end use of the microencapsulated particles (Li et al. 2010) and is an important research objective. For food fortification, it is important to consider the fact that food could be stored in humid places and must not release iron when inside the mouth, or during cooking etc. So, it is important to block the release of the iron when pH is around 7. The enteric coating material remains stable under high acidic condition of stomach (pH 1.5–3.5) for effective and tailored drug delivery to target organ (e.g., intestine) while reverse-enteric coating material readily releases its content at low pH (Dueik and Diosady 2016). Reverse-enteric materials can be used to develop a coating wherein iron would be protected when added into the food, but will be released in the stomach. Once the iron is released in stomach, it is available for absorption in intestines. Dueik and Diosady (2016) used chitosan and a pharmaceutical-grade polymer (Eudragit EPO, Evonik Industries) with this behavior. While chitosan is a linear polysaccharide that has numerous applications in the food industry due to its ready availability and biocompatibility, it is insoluble in water and organic solvents and is soluble in dilute aqueous acidic solutions. On the other hand, Eudragit EPO (Evonik Industries) is a cationic copolymer based on dimethylaminoethyl methacrylate, butyl methacrylate, and methyl methacrylate, which is soluble in gastric fluid up to pH 5.0. Eudragit EPO is available in a food-grade formulation known as Eudraguard. Its properties make it useful for moisture protection and taste masking. Both these coatings are hydrophobic at pH > 6.5 and hydrophilic at the gastric pH (< 2), which can potentially provide a good moisture barrier to the coated iron premix without hindering the gastric acid dissolution profile (Kwon 2005).

Various empirical and semi-theoretical models (Hanscomb and Kraft 2010; Sun et al. 2009) have been developed for investigating particle formation within spray dryers. However, no work has been conducted on modeling the release behavior of such food fortificants formed after spray drying. In this work, we have attempted to model the release behavior of reverse-enteric spray dried microcapsules under different pH conditions, to understand the inherent phenomena governing the release of such food fortificants. The iron fortificants prepared using chitosan and EPO were characterized and compared to assess their applicability for food fortification. To evaluate how perfectly the coating materials could encapsulate the iron salt, particle morphology and size distribution analysis was conducted using scanning electron microscopy (SEM). The release of iron was analyzed by simulating the conditions of the stomach and intestine and analyzing Fe using inductively coupled plasma spectrometry (ICP). The release profile was then fitted to various computational release models to gather understanding of the phenomenon responsible for iron release and approximate release times associated with each microcapsule prepared.

## Materials and Methods

### Materials

“Food-grade” ferrous sulfate heptahydrate (Fisher Scientific) was used as the iron source. Reverse-enteric coated iron microparticles were prepared using either medium molecular weight chitosan (Sigma Aldrich) or Eudagrit EPO (Evonik Industries) as coating agents. One percent acetic acid (Fisher Scientific) was used for enhancing the solubility of chitosan. Eudragit EPO formulation components were stearic acid (Sigma Aldrich), tartaric acid (BDH Chemicals), and talc (Sigma Aldrich). Stearic acid was used as it forms a soluble salt with Eudragit, allowing it to dissolve at high pH. Sodium lauryl sulfate in the standard pharmaceutical formulation (Dueik and Diosady 2016) was replaced by tartaric acid, which is listed as GRAS.

### Spray Solution Preparation

All experiments were carried out using distilled water. Chitosan feed solutions were prepared by dissolving 1% (*w*/*w*) of chitosan in 1% (*w*/*w*) acetic acid aqueous solution and left overnight for complete dissolution. Iron sulfate at 15, 20, 30, and 40% of the total weight of the solids was used to prepare microparticles of various iron loading. After dissolving ferrous sulfate for 15 min using a stirrer, the solution was spray dried as described in the “Spray Drying Conditions, Yield and Output” section.

Eudragit EPO is used in the pharmaceutical industry in formulations containing 15% total solids in water, with the composition of total solids being 57.1:5.7:8.6:28.5 for Eudragit EPO:tartaric acid:stearic acid:talc (Dueik and Diosady 2016). The spray solution was first prepared in two halves with talc dispersed in half of the water needed, and Eudragit EPO, tartaric acid, and stearic acid dissolved in the other half. The two liquids were mixed and homogenized for 90 min in a heavy duty laboratory mixer emulsifier (model L2R, Silverson, England). Ferrous sulfate at 10, 20, 30, and 40% of total solids was added and homogenized for 30 more minutes to prepare the feed solution for Eudragit microcapsules.

### Spray Drying Conditions, Yield, and Output

All samples were spray dried using a Buchi B290 mini-spray dryer (Buchi, Switzerland). Chitosan feed solutions were sprayed at 150 °C with a flow rate of 3 mL/min, an atomizing gas flow rate of 667 stdL/h at 618 kPa (90 psi) and an aspirator operating at − 4.5 Pa. In the case of Eudragit EPO solutions, the operating temperature was set to 110 °C, while other conditions were kept same. Later, dried microcapsules were collected from the bottom of the cyclone separator and were weighed to determine the process yield and process output. Process yield was defined as the percentage of the mass of iron premix produced to the mass of total solids in the liquid spray dried in a given time and is given by Eq. (1). Process output was defined as the ratio of the mass of the iron premix produced to the volume of the liquid spray dried and is given by Eq. (2).

1 $$ \mathrm{Yield}=\frac{\mathrm{mass}\  \mathrm{of}\  \mathrm{iron}\  \mathrm{microcapsules}\  \mathrm{collected}}{\mathrm{total}\  \mathrm{solids}\  \mathrm{in}\  \mathrm{the}\  \mathrm{spray}\  \mathrm{drying}\  \mathrm{solution}}\times 100\% $$

2 $$ \mathsf{Process}\mathsf{output}=\frac{\mathrm{mass}\  \mathrm{of}\  \mathrm{iron}\  \mathrm{microcapsules}\  \mathrm{collected}}{\mathrm{volume}\  \mathrm{of}\  \mathrm{the}\  \mathrm{liquid}\  \mathrm{spray}\  \mathrm{dried}} $$

### Morphology, Color, and Size of Microcapsules

Morphology of microcapsules was determined by scanning electron microscopy (SU-3500 VP SEM, Hitachi High-Technologies). The microcapsules were attached on SEM stub by carbon conductive double-coated adhesive tape and blast by air to remove any lose particle. Samples were examined and micrographs were recorded at an acceleration voltage of 1.5 kV, with working distance of 51 mm, under high vacuum. The size of the microcapsules was evaluated using image analysis of the micrographs using Matlab. The color of the microparticles was measured by taking high-quality photographs under white lightning and then analyzing the image using Matlab Image Analysis program.

### Total Iron Content and Iron Release Behavior

Total iron content of microcapsules was analyzed by using ICP-OES. Samples were first digested in microwave-assisted acid digestion system (MARS 6, John Morris Scientific Pty Ltd.) Briefly, 100 mg of microparticles was exposed to conc. HNO_3_ in a closed vessel and raising the temperature (200–210 °C) via microwave irradiation. Iron bound within the matrix solubilizes in clear digestate. The clear solutions obtained were quantitatively poured into a 25-ml flask and brought to volume with deionized water and further diluted with 5% nitric acid before analysis. Calibration curve was made with external iron by diluting a 1000 mg/L standard (Merck) solution.

In vitro iron bioavailability and iron release were analyzed according to a previously reported method (Swain et al. 2003; USP General Chapter 711 2011). Briefly, the rate of dissolution of iron in 0.1 N HCl solution (pH 1) was determined, because this solution resembles gastric juice. Iron release at pH 4 (0.0001 N HCl solution) was also used as it throws light on coating integrity (Romita et al. 2011). Iron release in phosphate buffer solution adjusted to pH 7 was also used in this study to estimate the amount of iron released by the microcapsules during food preparation and swallowing. The percentage of iron released in 30 min in pH 1 solution was considered to be in vitro bioavailability of microcapsules. Encapsulation efficiency, defined as the fraction of iron actually encapsulated in the microcapsule, was calculated by dissolving the microcapsules in pH 7 solution for 30 min and calculated using Eq. (3). Percentage of iron released in 1 h in pH 1 and pH 7 solution was used to calculate reverse enteric ratio (RE) according to Eq. (4):

3 $$ \%\mathsf{Encapsulation}\mathsf{efficiency}=\frac{\mathsf{totalironcontent}-\mathsf{unencapsulated}\mathsf{iron}\mathsf{released}\ \mathsf{at}\ \mathsf{pH}\ \mathsf{7}}{\mathsf{totalironcontent}}\times \mathsf{100}\% $$

4 $$ \mathsf{Reverse}\mathsf{enteric}\mathsf{ratio}\ \left(\mathsf{RE}\right)=\left(\frac{{\mathsf{release}}_{\mathsf{pH}\kern0.1em \mathsf{1}}}{{\mathsf{release}}_{\mathsf{pH}\kern0.1em \mathsf{7}}+{\mathsf{release}}_{\mathsf{pH}\kern0.1em \mathsf{4}}}\right) $$

Approximately 20 mg of each spray dried powder was weighed and dispersed into three 250-ml volumetric flasks, containing 200 ml each of pH 1 HCl solution, pH 4 HCl solution, and 200 pH 7 phosphate buffer solution, respectively. The flasks were then placed in a shaking water bath set at 37 °C for 2 hours. Five milliliter aliquots from each flask were diluted to 25 ml in volumetric flasks at various intervals (15, 30, 60, 90, and 120 min) with 5% nitric acid. The iron content in the samples was measured using ICP-AES.

### Models for Iron Release from Microcapsules

Iron release from these microcapsules was fitted to the five most common models (Costa and Lobo 2001). A zero-order model describing the linear fitting between the release time curves was obtained using Eq. (5):

5 $$ \mathit{\mathsf{Y}}={\mathit{\mathsf{K}}}_{\mathsf{0}}\mathit{\mathsf{t}}+{\mathit{\mathsf{C}}}_{\mathsf{0}} $$

where *Y* is the percentage (*w*/*w*) of iron released at any specific time *t*, and *K*_0_ and *C*_0_ are the slope and intercept of the *y*-*t* curve.

A first-order model was fitted by plotting the natural logarithm of the percentage of iron left in the encapsulated matrix with time, expressed by Eq. (6):

6 $$ \mathsf{\ln}\left(\mathsf{100}-\mathit{\mathsf{Y}}\right)={\mathit{\mathsf{K}}}_{\mathsf{1}}\mathit{\mathsf{t}}+{\mathit{\mathsf{C}}}_{\mathsf{1}} $$

where *Y* is the percentage (*w*/*w*) of iron released at any specific time *t*, and *K*_1_ and *C*_1_ are slope and intercept of the ln *y* vs. *t* curve.

A general empirical equation described by Weibull (1951) was adapted to the release process (Langenbucher 1972). The adapted Weibull model can be expressed using Eq. (7):

7 $$ \log \left[-\ln \left(\mathsf{1}-\frac{\mathsf{Y}}{\mathsf{100}}\right)\right]=\mathsf{b}\log \mathsf{t}-\log \mathsf{a} $$

where *Y* is the percentage (*w*/*w*) of iron released at any specific time *t*, and *b* and − log *a* are slope and intercept of the log [− ln(1 − *Y*/100)] vs. log *t* curve.

Higuchi (1963) model, describing a linear relationship between the percentage of release of the iron and square root of time, was represented by Eq. (8):

8 $$ \mathit{\mathsf{Y}}={\mathit{\mathsf{K}}}_{\mathit{\mathsf{h}}}{\mathit{\mathsf{t}}}^{\mathsf{0.5}} $$

where *Y* is the percentage (*w*/*w*) of iron released at any specific time *t*, and *K*_*h*_ is the slope of the log *Y* vs. *t* ^0.5^ curve.

Hixson and Crowell (1931) model was used to depict the condition where the cubic root of the iron left in the matrix was linear with respect to time. Equation (9) was used to model this scenario:

9 $$ {\left(\mathsf{100}-\mathit{\mathsf{Y}}\right)}^{\mathsf{1}/\mathsf{3}}={\mathit{\mathsf{K}}}_{\mathit{\mathsf{s}}}\mathit{\mathsf{t}}+{\mathit{\mathsf{C}}}_{\mathit{\mathsf{s}}} $$

where *Y* is the percentage (*w*/*w*) of iron released at any specific time *t*, and *K*_*s*_ and *C*_*s*_ are the slope and intercept of the (100 − *Y*)^1/3^ vs. t curve.

### Statistical Analysis

Microsoft Excel (Ver. 15.33, Microsoft Excel for Mac, Microsoft) was used for regression. Matlab was used for image analysis of the SEM micrographs. The *R* ^2^ of the various fitting equations were compared to assess the significance of fit. Student’s *t* test was conducted for comparing two sets of data to determine if they were significantly different from each other. All experimental results were expressed as mean ± standard deviation.

## Results and Discussions

### Process Yield

The yield of a spray drying process is an important indicator for the economic feasibility and scalability of the process. The process yield and process output for the preparation of the various microcapsules are presented in Table 1. The data indicate that, although the yield from chitosan and Eudragit was in the same range (65–75%), the process output from Eudragit was almost 10 times higher than that from the chitosan samples. This is due to the fact that the total solids in the Eudragit spray solution were much higher (~ 15–20%) than those in the chitosan spray solution (1–2% total solids) due to its higher viscosity. This effectively translates into a better industrial process economy while using Eudragit as the coating medium, as less solution has to be processed during the drying process, leading to 10 times lower processing times. In other words, spray drying equal volumes of Eudragit and chitosan solutions will yield 10 times more products from the former.

**Table 1:** Characteristics of the various microcapsules prepared

Sample	Process yield (%)	Process output (g/l)	L*	a*	b*	Iron Bioavailability (%)	Encapsulation efficiency (%)	Reverse entericity ratio (RE)
Chitosan 15%	72^1^	16.6^1^	64.1^1^	7.4^1^	29.6^1^	89^1^	98^1^	1.3^1^
Chitosan 20%	75^2^	18.0^2^	58.4^2^	12.3^2^	38.6^2^	69^2^	95^2^	1.5^2^
Chitosan 30%	68^3^	17.7^2^	57.7^2^	15.6^2^	39.2^2^	87^1^	91^3^	1.0^3^
Chitosan 40%	74^2^	20.7^3^	51.7^2^	16.4^2^	44.3^3^	93^3^	95^2^	1.0^3^
Eudragit 10%	75^2^	123.7^4^	94.3^3^	−4.2^3^	33.5^4^	94^3^	91^3^	1.5^2^
Eudragit 20%	73^1^	131.4^5^	87.1^3^	4.1^1^	41.1^3^	82^4^	87^4^	1.0^3^
Eudragit 30%	68^3^	132.6^5^	71.6^4^	10.5^1^	43.7^3^	93^3^	85^4^	1.2^3^
Eudragit 40%	74^2^	155.4^6^	62.9^1^	15.3^2^	46.4^4^	99^5^	94^2^	2.4^4^

Same values in superscript within a column indicate groups of means which are not statistically different from each other according to Duncan’s multiple range test

A similar yield of 72% was reported for chitosan in a previous study (Dueik and Diosady 2016). The losses in both our study and Dueik and Diosady (2016) are mainly on account of these iron capsules sticking to the instrument wall or passing through the filter. Amiri-rigi et al. (2011) mentioned that the high total electric charge formed during the formation of the microcapsules is responsible for the losses to the instrument wall. Moreover, lightweight particles are also lost due to the suction created by the vacuum pump and could only be collected from the air filter of the vacuum pump. An analysis of these fines revealed that these powders were an order of magnitude smaller in size than the main product (size of the microcapsules is provided in the “Morphology of the Prepared Microcapsules” section).

### Morphology of the Prepared Microcapsules

Table 1 presents the L*, a*, and b* of the prepared microcapsules after the spray drying process. The color of the microcapsules became browner, with more darkness (lower L* value), redness (higher a* value), and yellowness (higher b* value), on increasing the iron loading. In both cases, higher iron loading results in darker premixes. It is noteworthy that the color of the iron premix often has to be controlled depending on the purpose of iron fortification. Some formulations, such as those for fortification of salt or rice, require a whiter premix, whereas formulations, such as those for tea or coffee fortification, might prefer a darker brown premix. Our premix was more suitable for the latter formations. However, there is a possibility of using a small amount of colorant to lighten the color of the premix. In such cases, the L*a*b* data in Table 1 could provide guidance to the amount of colorants needed for matching the color of the product.

**Fig. 1:**
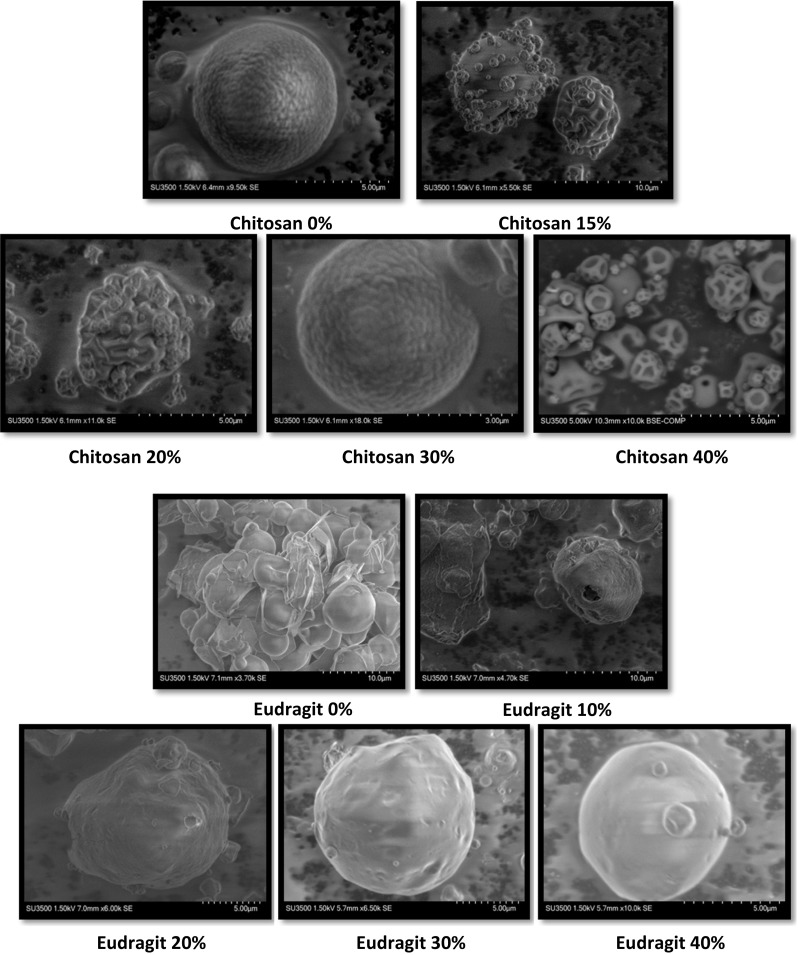
SEM images of the prepared microcapsules (magnification shown in image)

As seen in the SEM images (Fig. 1), the uniformity of coating for chitosan microcapsules increased as the amount of iron was increased from 15 to 20 to 30%; however, at 40% iron loading, the coating disintegrated with a significantly reduced size of the microcapsule. On the other hand, Eudragit microcapsules formed the best microcapsules at 40% iron loading and the uniformity of coating increased on increasing iron loading. The average size of the microcapsules was evaluated using image analysis of these SEM images and varied between 3 and 5 μm, with no significant difference (*p* > 0.05) between the sizes of the microcapsules of various formulations, except chitosan 40%, whose microcapsules were between 0.5 and1.5 μm and were significantly (*p* < 0.05) smaller than the others. The smaller size of the microcapsules at 40% ferrous sulfate loading, are indicative of the fact that these microcapsules due to the low amount of chitosan (1% *w*/*w* solids) present relative to the amount of iron, in contrast to high amount (22% *w*/*w* total solids) of coating material in Eudragit-based microcapsules. At this high level of encapsulate loading, the encapsulant was probably insufficient to provide a good film, and hence, there was no impedance to mass transfer and the particles kept on shrinking further, resulting in smaller particles with imperfect capsules (Amiri-rigi et al. 2011). From these results, it can be concluded that chitosan microcapsules with 30% ferrous sulfate loading and Eudragit with 40% ferrous sulfate loading resulted in producing desirable microcapsules for our purposes with higher iron loading and particle uniformity.

### Iron Content, Iron Bioavailability, Encapsulation Efficiency, and Reverse-Enteric Behavior

**Fig. 2:**
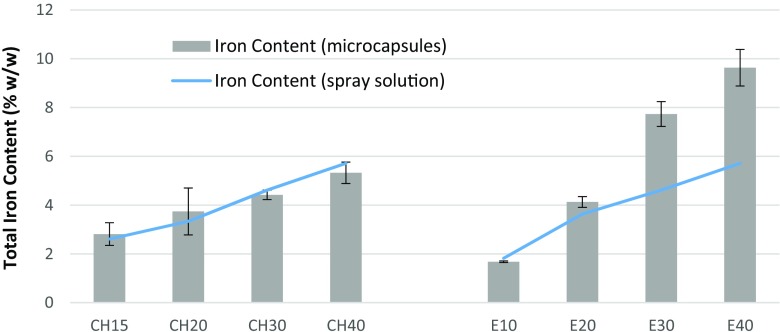
Total iron content (%*w*/*w*) in the spray solution and in the iron microcapsules prepared after spray drying

Spray drying as a technology involves rapid evaporation of small droplets. The concentration of the various constituents in the dried powder may change, depending on the flow rate of the aspirator, the efficiency of the cyclone separator, and other process parameters. Therefore, it is important to investigate the iron content in the spray solution before and then in the prepared microcapsules after spray drying. Figure 2 shows the total iron content of the spray solutions and the prepared microcapsules. There was no significant difference (*p* > 0.05) between the means of the iron content of the microcapsules and spray solution for chitosan. This is expected, if all of the spray solution is dried to form particles of similar composition. However, for Eudragit, it was seen that there was a significant difference (*p* < 0.05) in the iron content of the prepared microcapsules with increase in iron loading. This could be explained by the non-homogenity during the spray drying process. The lighter microcapsules formed with low iron content escaped the system through the air exhaust, leaving particles collected at the bottom enriched in the heavier iron. Figure 2 also suggests that Eudragit microencapsulation proved efficient as microcapsules with up to 9.6% *w*/*w* iron content could be obtained, which was less than those created earlier in our group (Li et al. 2010; Romita et al. 2011) using macroencapsulation technology of agglomeration and extrusion processes (which produce microcapsules with around 16–18% *w*/*w* iron content), but might still be acceptable as a microencapsulation approach. As seen in the “Morphology of the Prepared Microcapsules” section, the uniformity of encapsulation was also better for Eudragit microcapsules. The advantage of the Eudragit microcapsules is also further enhanced by the better process output of Eudragit due to the significantly higher total solid content (about 20 times) of the Eudragit spray solutions than that of the chitosan solution, as explained in the “Process Yield” section.

**Fig. 3:**
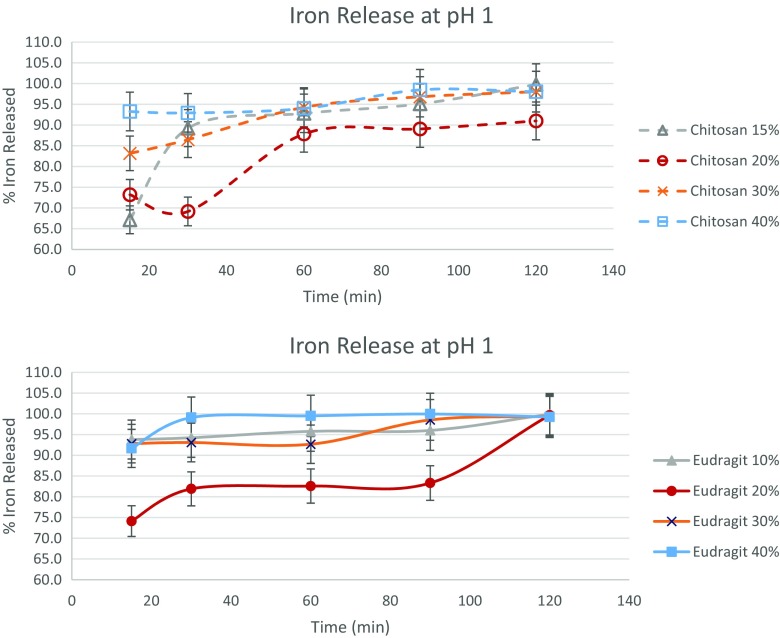
Iron released from the various chitosan and Eudragit microcapsules at pH 1 as measured via release in 0.1 N HCl

Figure 3 shows the percentage of iron release from the microcapsules in pH 1 HCl solution. This pH is often used in in vitro bioavailability studies, as it approximates the availability of iron inside the stomach. Most of the microcapsules released almost 100% of the iron present in the samples within 2 h at pH 1. In general, chitosan microparticles exhibited faster release than Eudragit microcapsules. More than 90% of the iron was released within 15 min for all microcapsules, except those with 20% ferrous sulfate loading. On the other hand, for chitosan, only “Chitosan 40%” could achieve more than 90% iron release. There was a delay between 15 and 30 min for release of 90% iron for the chitosan microcapsules, as compared to the Eudragit under stomach conditions. While this delayed release could be a property of choice for some specific applications, a faster availability in pH 1 solution is generally desirable for food iron fortification initiatives, with moderate pH levels. In vitro bioavailability of the samples was analyzed by calculating the percentage of iron released after 30 min, which is the approximate minimal time spent by food in the stomach. For Eudragit samples, iron was almost completely bioavailable (> 90%) for all samples, except at 20% ferrous sulfate loading with 82% bioavailability. On the other hand, for chitosan samples, the iron bioavailability was 89, 69, 87, and 93% for 15, 20, 30, and 40% ferrous sulfate loading, respectively. In general, the bioavailability of iron was higher for Eudragit samples.

**Fig. 4:**
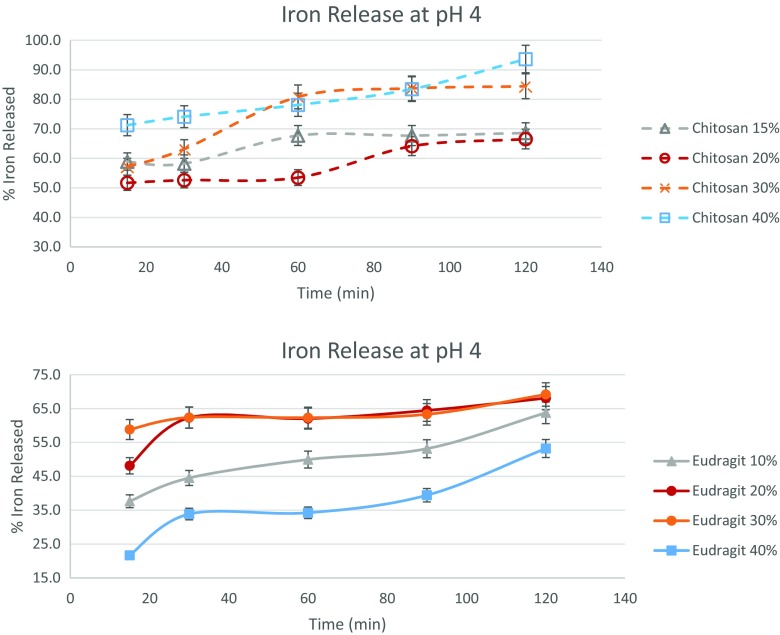
Iron released from the various chitosan and Eudragit microcapsules at pH 4 as measured via release in 0.0001 N HCl

Figure 4 details the pH release behavior in pH 4 solution. The iron release in pH 4 is generally used to check the stability of the coating for iron that is insoluble at pH 7, but might still be released under conditions which are less acidic. Under mild stirring at pH 4, as much as 67–94% of iron was released in 2 h for chitosan microcapsules, as compared to 53–70% iron released for Eudragit encapsulated particles. Thus, it is seen that for comparing the coating integrity, Eudragit microcapsules offer better protection than their chitosan counterparts. Forty percent Eudragit encapsulated particles, with only 53% iron released, was the most effective coating tested.

**Fig. 5:**
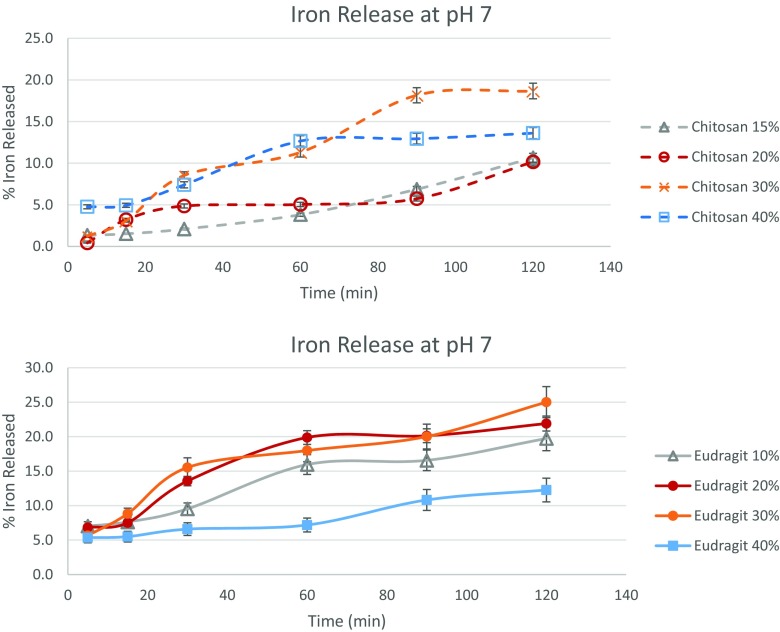
Iron released from the various chitosan and Eudragit microcapsules at pH 7 as measured via release in a phosphate buffer of pH 7

Figure 5 depicts the release behavior in pH 7 solution. This represents the amount of iron released during soaking/cooking/processing of food in water. It must be mentioned that this release behavior does not take into account the effect of temperature. Human saliva has a healthy pH around 7.4, but this can vary from 5.9–7.9 (Feller and le Petit 1977; Ritschel and Thompson 1983). Thus, iron release in neutral pH also approximates the amount of iron that would be released in the mouth. However, any release of iron before swallowing will directly affect the taste of the food. Only 10–18% of the iron in chitosan microencapsulated particles was released with gentle shaking in a pH 7 solution for 2 h. On the other hand, for Eudragit particles, 12–25% iron particles were released in the same time. It is notable that Eudragit microparticles with 40% ferrous sulfate loading gave the lowest iron release, 12% at pH 7 depicting better coating integrity of these microparticles. Although, the best protection against neutral pH conditions was achieved by chitosan 15 and 20% iron loaded microcapsules.

As a result of the observations for pH 7, the encapsulation efficiency of chitosan microcapsules (91–97%) was found better than that of Eudragit microcapsules (85–94%) (Table 1). This suggests the superior protection offered by chitosan microcapsules under neutral pH conditions. However, to be used as a reverse-enteric coating material, release at pH 1 is also an important factor to consider to ensure high bioavailability. As shown in Figs. 3, 4, and 5, the amount of the iron released from these microcapsules was high at pH 1 and then decreased as the pH increased from 1 to 7, as expected with reverse-enteric coating. This behavior is optimal for iron release and absorption. The reverse entericity (RE) ratio of the various microcapsules were computed based on release after 1 h and presented in Table 1. Eudragit 40% microcapsules had the maximum RE ratio (2.4). Amongst the chitosan microcapsules, 20% ferrous sulfate loading chitosan microcapsules had the maximum RE ratio (1.5), again confirming that Eudragit at 40% was the most effective coating tested.

### Release Parameters for the Prepared Microcapsules

It is important to characterize the release behavior by fitting it to well-known models in the literature to provide data for mathematical modeling of the release behavior of our iron microcapsules. We fitted the obtained release profile at various pH for these iron microcapsules for a period of 2 h with various models, and the fitted parameters are provided in Table 2. Nevertheless, the zero-order and first-order models are the most common and well-understood models used for modeling in chemical engineering investigations (Kitazawa et al. 1977; Mulye and Turco 1995); other more sophisticated models can throw a greater light into the active mass transfer phenomenon. Thereby, Higuchi model (Higuchi 1963), Hixson-Crowell model (Hixson and Crowell 1931), and Weibull Model (Langenbucher 1972) were also tested.

**Table 2:** Parameters and Determination Coefficients of the various models tested

Model		Zero order			First order			Higuchi		Hixson-Crowell			Weibull Model		
	pH	*K*_0_	*C*_0_	*R* ^2^	*K*_1_	*C*_1_	*R* ^2^	*K*_*h*_	*R* ^2^	*K*_*s*_	*C*_*s*_	*R* ^2^	*b*	*a*	*R* ^2^
Chitosan 15%	1	0.25	73.38	0.69	0.040	4.10	0.84	10.99	0.95	0.021	3.23	0.89	0.68	5.40	0.88
	4	0.11	57.53	0.78	0.003	3.75	0.79	7.83	0.93	0.003	3.49	0.78	0.15	1.77	0.83
	7	0.08	0.12	0.95	0.001	4.60	0.95	0.72	0.91	0.001	4.64	0.95	0.66	307.74	0.86
Chitosan 20%	1	0.21	68.98	0.78	0.012	3.52	0.85	10.11	0.94	0.010	3.19	0.83	0.35	2.20	0.81
	4	0.16	47.93	0.89	0.004	3.97	0.89	7.11	0.95	0.004	3.75	0.89	0.20	2.52	0.75
	7	0.07	1.43	0.84	0.001	4.59	0.85	0.77	0.96	0.001	4.62	0.85	0.85	556.28	0.85
Chitosan 30%	1	0.15	82.58	0.90	0.021	3.14	0.99	11.17	0.93	0.013	2.71	0.97	0.40	1.76	0.97
	4	0.27	56.78	0.83	0.010	3.82	0.87	9.25	0.96	0.010	3.55	0.86	0.42	3.81	0.95
	7	0.16	1.74	0.94	0.002	4.59	0.95	1.64	0.97	0.003	4.62	0.94	0.92	340.47	0.98
Chitosan 40%	1	0.06	91.74	0.83	0.015	2.30	0.77	11.45	0.91	0.008	2.09	0.79	0.22	0.73	0.68
	4	0.20	67.41	0.96	0.013	3.70	0.86	9.86	0.94	0.011	3.31	0.90	0.32	2.13	0.76
	7	0.09	4.81	0.87	0.001	4.56	0.87	1.38	0.99	0.001	4.57	0.87	0.41	46.37	0.90
Eudragit 10%	1	0.05	92.64	0.87	0.033	2.90	0.65	11.51	0.91	0.011	2.17	0.72	0.32	0.98	0.51
	4	0.22	35.78	0.96	0.005	4.19	0.95	6.28	0.97	0.006	4.03	0.95	0.33	5.22	0.93
	7	0.12	6.59	0.95	0.001	4.54	0.95	1.86	0.99	0.002	4.54	0.95	0.37	29.43	0.90
Eudragit 20%	1	0.19	72.18	0.78	0.034	4.27	0.62	10.34	0.94	0.017	3.38	0.68	0.49	3.17	0.52
	4	0.14	51.88	0.67	0.004	3.88	0.72	7.51	0.94	0.004	3.64	0.70	0.23	2.57	0.82
	7	0.14	7.66	0.86	0.002	4.53	0.87	2.20	0.99	0.002	4.52	0.87	0.45	32.70	0.93
Eudragit 30%	1	0.07	90.85	0.81	0.025	2.72	0.85	11.46	0.91	0.011	2.26	0.84	0.30	0.99	0.65
	4	0.08	58.23	0.82	0.002	3.74	0.81	7.65	0.92	0.002	3.48	0.81	0.10	1.50	0.73
	7	0.15	7.40	0.91	0.002	4.53	0.92	2.30	0.99	0.003	4.53	0.92	0.49	37.70	0.97
Eudragit 40%	1	0.05	94.60	0.41	0.027	1.26	0.37	11.76	0.91	0.010	1.61	0.44	0.40	0.99	0.64
	4	0.25	20.81	0.88	0.004	4.40	0.87	4.74	0.98	0.005	4.31	0.88	0.45	13.19	0.87
	7	0.06	4.60	0.95	0.001	4.56	0.95	1.13	0.97	0.001	4.57	0.95	0.27	32.87	0.80

The mean of the *R* ^2^ for the fit between the zero-order and first-order models ranged around 0.84 ± 0.12 and 0.83 ± 0.14, respectively, suggesting that neither could produce a fit, but, as these *R* ^2^ are within acceptable range of > 0.7, they could be used for simpler mathematical modeling purposes. Although, this demonstrates that both zero- and first-order models are equally acceptable, the values of the release predicted at *t* = 0 is given by the constants *C*_0_ and *C*_1_. While *C*_1_ ranged between 3.8 ± 0.8, *C*_0_ showed a greater variability with their means around 45.7 ± 34.6. Thus, it can be concluded that the first-order model was a better choice than the zero-order model, as the release at *t* = 0 should not vary much from 0. This is also evident in the general applicability scenarios for these models. Whereas the zero-order model is more applicable to pharmaceutical dosage forms that do not disaggregate and release the drug slowly (Varelas et al. 1995), the first-order model is applicable in the case of porous drug matrices. Our system is closed to a first-order model, where the amount of drug released is proportional to the amount of drug left over in the interior and decreased with time.

Amongst all the models tried in this study, Higuchi model gave the best fit (mean *R* ^2^ = 0.95 ± 0.03). Mean *R* ^2^ for the Hixson-Crowell and Weibull models ranged around 0.84 ± 0.12 and 0.81 ± 0.13, respectively, and were similar to that for the first-order model. Yet, in terms of the absolute value of the means, the fitting of the models may be arranged as follows: Higuchi > Hixson-Crowell > first order > Weibull. Thus, Higuchi (1963) model was found to be the best fit iron release data from our system. Higuchi model has been traditionally developed to study the release of water-soluble and low-soluble drugs incorporated in semi-solid and/or solid matrices. This model describes the drug release as a diffusion process described by Fick’s law, with the dissolution dependent on the square root of the time. Higuchi model has been used in many pharmaceutical systems such as those in the case of transdermal systems (Costa and Lobo 2001) and in matrix tablets with water-soluble drugs (Schwartz et al. 1968). Our iron fortification premix coated with chitosan and Eudragit polymers resembles the latter case, iron sulfate being the water-soluble drug in our study. Being based on Fick’s law, square of the Higuchi dissolution constant (*K*_*h*_) is directly related to the diffusivity from the matrix (Costa and Lobo 2001) and can be used for such calculations. Obviously, the relation between the dissolution constant and the diffusivity also depends on the surface properties of the material including shape, size, and sphericity. Based on the values of *K*_*h*_, it can be concluded that, as compared to chitosan, Eudragit samples offered better diffusivity under stomach conditions at pH 1 (mean *K*_*h*_ = 10.93 ± 0.58 for chitosan and mean *K*_*h*_ = 11.27 ± 0.63 for Eudragit), and lower diffusivity at pH 4 (mean *K*_*h*_ = 8.51 ± 1.26 for chitosan and mean *K*_*h*_ = 6.55 ± 1.35 for Eudragit). However, at pH 7, which is important for the taste masking behavior, chitosan provided a better impediment to iron release (mean *K*_*h*_ = 1.13 ± 0.46 for chitosan and mean *K*_*h*_ = 1.87 ± 0.53 for Eudragit). Yet, it must be mentioned that Eudragit with 40% loading had a *K*_*h*_ = 1.13 at pH 7, which was same as that for chitosan. Thus, overall, Eudragit 40% is found to be a better choice for food fortification purposes. These calculations confirm results found in the previous sections.

It is important to understand that the high *R* ^2^ associated with Higuchi model cannot be used for comparison with other models. Higuchi model is a constrained model with only one parameter *K*_*h*_ represented by the slope of the (*y*-*t* ^0.5^) curve, whereas, other models have two-parameters with the slope of the curve yielding the value of one parameter (*K*_0_, *K*_1_, *K*_*s*_, *b*) and the constant yielding the value of the other parameter (*C*_0_, *C*_1,_ *C*_s_, −log *a*). Due to this, essentially during curve fitting with excel, during Higuchi model, the constant is forced to 0, yielding a higher emphasis on the origin points (*y* = *t* = 0). Under these circumstances, *R* ^2^ is computed with a slightly different formula, due to which a higher value of *R* ^2^ is obtained. Hence, it is important to check other models as well. Weibull model fitted the worst amongst the models compared, yet its *R* ^2^ was only slightly lower than other models, and this model could still be considered acceptable for deriving meaningful deductions. Since, the b* values of the model were less than 1 in our case, the dissolution profile can be understood to be parabolic, with a higher initial slope, and after that consistent with exponential profile. Yet, again, for in vitro studies, Weibull model has been under severe criticism (Christensen et al. 1980; Pedersen and Myrick 1978) as it could never adequately characterize the dissolution kinetic properties of the drug due to the absence of any single parameter related to the dissolution rate of the drug. Our lower *R* ^2^ values can be attributed to this phenomenon. Hixson-Crowell model was a better fit as compared to Weibull model, while its fit to the Higuchi model could not be compared. Hixson and Crowell (1931) model assumes that the release rate is limited by the drug particle dissolution rate and not diffusion process through the matrix. The applicability of both Higuchi and Hixson-Crowell models suggests that both the mechanisms of slowed release due to diminishing surface of the drug particles during the dissolution and the diffusion process through the barrier created by the coating material are of concern for modeling the release behavior of microencapsulated iron microparticles.

## Conclusions

This study presents the possibility of using spray drying for the purposes of food fortification. Spray drying-based encapsulation was attempted using two coating materials and their iron release was modeled with various well-known dissolution models. Higuchi model was found best to model the iron release behavior of these spray drying encapsulated microcapsules. The applicability of Higuchi and Hixson-Crowell models suggests a transport phenomenon limited both by the dissolution and diffusion processes. Spray drying encapsulated microcapsules, particularly the Eudragit encapsulated microcapsule with 40% iron loading, could provide up to 9.6% *w*/*w* of iron, which the same order of magnitude as those produced by extrusion processes. All samples had desirable bioavailability, high encapsulation efficiency, good coating integrity, and low release in neutral pH. Eudragit encapsulated microcapsules with 40% ferrous sulfate loading and chitosan encapsulated microcapsules with 30% ferrous sulfate loading were found to be the best candidates for possible application in future research. It is noteworthy that the Eudragit coating, due to higher solid content, can handle higher amount of iron payload. Despite its competitive properties and better encapsulation efficiency than Eudragit, process yield of Chitosan was low, due to the relatively high viscosity at low concentration and low solid content, suggesting a possibility of exploring secondary polymers with chitosan in future work. The new methodology developed for studying the iron release of microencapsulated microparticles could be used in future applications in food fortification research.
